# Sequences conserved by selection across mouse and human malaria species

**DOI:** 10.1186/1471-2164-8-372

**Published:** 2007-10-15

**Authors:** Hideo Imamura, Jason H Persampieri, Jeffrey H Chuang

**Affiliations:** 1 Boston College – Department of Biology, 140 Commonwealth Avenue, Chestnut Hill, MA 02467, USA. Phone: 617-552-0804

## Abstract

**Background:**

Little is known, either experimentally or computationally, about the genomic sequence features that regulate malaria genes. A sequence conservation analysis of the malaria species *P. falciparum*, *P. berghei*, *P. yoelii*, and *P. chabaudi *could significantly advance knowledge of malaria gene regulation.

**Results:**

We computationally identify intergenic sequences conserved beyond neutral expectations, using a conservation algorithm that accounts for the strong compositional biases in malaria genomes. We first quantify the composition-specific divergence at silent positions in coding sequence. Using this as a background, we examine gene 5' regions, identifying 610 blocks conserved far beyond neutral expectations across the three mouse malariae, and 81 blocks conserved as strongly across all four species (p < 10^-6^). Detailed analysis of these blocks indicates that only a minor fraction are likely to be previously unknown coding sequences. Analogous noncoding conserved blocks have been shown to regulate adjacent genes in other phylogenies, making the predicted blocks excellent candidates for novel regulatory functions. We also find three potential transcription factor binding motifs which exhibit strong conservation and overrepresentation among the rodent malariae.

**Conclusion:**

A broader finding of our analysis is that less malaria intergenic sequence has been conserved by selection than in yeast or vertebrate genomes. This supports the hypothesis that transcriptional regulation is simpler in malaria than other eukaryotic species. We have built a public database containing all sequence alignments and functional predictions, and we expect this to be a valuable resource to the malaria research community.

## Background

Malaria is a disease caused by parasites in the genus *Plasmodium*. It afflicts 500 million people worldwide and kills more than one million each year [1]. In the last few years, significant progress has been made towards characterizing the molecular biology of malaria, including the sequencing of the genomes of *P. falciparum *and several other malaria species [2]. While this sequencing has led to the identification and characterization of many malaria genes, little is known about the transcription factor binding sites or other sequence features that are involved in gene regulation. Only a handful of gene regulatory interactions have been characterized experimentally [3-8] (reviewed in [9]) – such studies are generally more difficult than in comparatively facile models such as yeast. A complementary and alternative approach to bench experiments is a computational comparative genomic analysis. Cross-species sequence comparisons can provide a great deal of information about potential functional sites, as has been shown in other phylogenies [10-12]. Specifically, the extensively sequenced genomes of *P. falciparum *[2] and three related mouse malaria species, *P. berghei*, *P. yoelii*, and *P. chabaudi *[13-15], are a rich resource for identifying novel functional sites in malaria.

Novel computational studies of gene regulation may be particularly valuable for malaria. It has been speculated that the malaria gene regulatory program is simpler than that of most eukaryotic organisms, due to the small number of proteins homologous to known transcription factors in other species [16] and the relative homogeneity of *P. falciparum *mRNA expression patterns [17,18]. A few such computational studies have been performed. Militello et al [3] identified a GC-rich motif (the G-box) occurring in the sequences 5' of several *P. falciparum *genes and their counterparts in *P. yoelii*, *P. berghei*, and *P. vivax*, and verified the activity of this sequence experimentally. van Noort and Huynen [19] predicted 12 motifs based on correlation of unaligned *P. falciparum *and *P. yoelii *promoter sequences with mRNA expression data. Similarly, multiple groups have used protein/mRNA ratios to identify motifs likely to affect mRNA degradation, with varying levels of confidence [14,20]. As in the Militello et al work, sequence conservation provided support for one of these motifs [14]. However, a more thorough sequence conservation analysis, involving comprehensive alignments and conservation measurements, has the potential to be much more powerful. Conservation may reveal functional sequences throughout all alignable regions of the genome, rather than merely next to genes with particular expression patterns.

**Figure 1:**
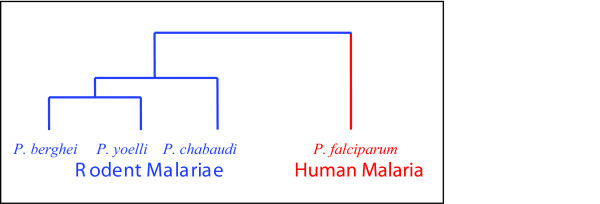
**Phylogeny of Rodent and Human Malariae**. The three mouse malaria species, *P. chabaudi*, *P. berghei*, and *P. yoelii*, are closely related, with 0.757 of the alignable 4-fold sites identical across all three mouse species. *P. falciparum *has saturated divergence from these three, with 0.359 of the alignable 4-fold sites identical across all four species. AT sites are much more commonly identical than GC sites in each grouping.

In this paper we therefore undertake a large scale sequence conservation analysis, computationally identifying intergenic sequences conserved beyond neutral expectations across several malaria genomes. This technique, commonly referred to as phylogenetic footprinting, has been shown to effectively detect functional regions in yeast and vertebrate phylogenies[11,12,21]. Our work makes use of publicly available sequence data from the genomes of the human malaria *P. falciparum *and the rodent malariae *P. berghei*, *P. chabaudi*, and *P. yoelii *(Figure 1). Neafsey et al considered a similar paradigm in a comparison of *P. falciparum *to the chimpanzee malaria *P. reichenowi *[22]. Because of the extreme sequence identity of *P. falciparum *and *P. reichenowi *(>90%), these genomes are not well-suited for distinguishing specific sequences under selective constraint. However, Neafsey et al did observe an unusual excess of CpG sites in intergenic regions proximal to genes, suggesting that these regions contain functional sequences. Because our study analyzes species at greater phylogenetic separations, we are able to refine this approach to identify specific locations likely to be functional.

In this work, we first quantify the neutral divergence between the species, using silent positions in coding sequence. The statistics of silent sites provide a background for evaluating conserved intergenic sequences of differing lengths and base composition. Composition is a crucial consideration in malaria, since the genome has a strong bias for AT, with *P. falciparum *having 81% AT content [15].

Given this neutral background, we take two approaches to identifying sequences conserved by selection. In the first, we determine contiguous blocks of intergenic sequence which are conserved beyond the neutral background, and which are therefore presumably functional. We consider the total amount of such unusually conserved sequence and analyze the genes next to which these regions occur. We are able to identify 610 blocks conserved strongly across the three mouse malariae, and 81 blocks conserved strongly across all four species including *P. falciparum*.

In the second approach, we identify sequence motifs whose instances are conserved more frequently than random sequences. Such motifs are good candidates for transcription factor binding sites, which typically occur in each of the promoters of co-regulated genes. We focus on three motif families with compelling evidence for functionality. The motifs in each of these families are highly conserved and occur much more often than would be expected by chance.

Our phylogenetic footprinting findings provide a valuable new resource for understanding functional intergenic sequences in malaria. Given the small number of known regulatory sequences in malaria, our predictions provide a substantial improvement to the knowledge in this area. To ensure the usefulness of our findings to others in the field, we have made all of the alignments and predictions available in a user-friendly website [see Additional file 1].

## Results

### Malaria Species Divergence

To estimate the background neutral conservation level, we calculated the substitution rate in silent positions in aligned orthologous coding sequence, in particular 4-fold degenerate sites. Silent sites, such as 4-fold degenerate sites, have conventionally been chosen as a good proxy for neutral sequence [23], which is why we have chosen to use them to determine the neutral background in our study. Previous studies in yeast [10] also suggest that the selective pressures on 4-fold sites are small enough that they are a reasonable approximation to neutrality (see Discussion). We observed 4-fold site substitution rates (the fraction of 4-fold sites in which the bases differ between the species) of 0.121 (*P. berghei*-*P. yoelii*), 0.191 (*P berghei*-*P. chabaudi*), and 0.194 (*P. yoelii*-*P. chabaudi*). These divergences are qualitatively consistent with the maximum-likelihood inferences reported by Hall et al, who found median dS values of 0.03 (*P. berghei*-*P. yoelii*), 0.5 (*P. berghei*-*P. chabaudi*), and 0.5 (*P. yoelii*-*P. chabaudi*). The quantitative differences are due to their use of a heterogeneous codon-based approach, as well as the multiple substitutions allowed in a maximum likelihood formulation. For the three mouse malaria species, 0.757 of the 4-fold sites are identical across all three mouse species. This leads to the rough expectation that random intergenic regions of length N should be identical with a probability 0.757^N^, if one were to ignore alignability or base composition considerations. Under this assumption a block needs to be of length ~50 to have a conservation p-value of 10^-6^. In practice, the strong AT bias in malaria species allows blocks of shorter length to be identified. Identical GC bases are rare in 4-fold sites, which increases their significance for discriminating unusual blocks. Overall, the fraction of 4-fold sites which are an identical A or T is 0.694, the fraction which are an identical G or C is 0.063, and the fraction which are not identical is 0.243. We use these values to assess unusually conserved regions.

The fraction of *P. falciparum *4-fold sites identical across all 4 species is 0.359, which yields an estimate that conserved sequences must be ~13 bases long for detection at p-value 10^-6^. The fraction of 4-fold sites which are an identical A or T is 0.3504, the fraction which are an identical G or C is 0.0085, and the fraction which are not identical is 0.6411. Hall et al reported that all three mouse species have saturated neutral divergence with respect to *P. falciparum*. In our dataset, the fraction of 4-fold sites identical between *P. yoelii *and *P. falciparum *is 0.441. This is close to, but slightly higher than what would be expected (0.379) if the 4-fold bases have no remnants of shared history and are not under selection, given each species' 4-fold base compositions. The higher observed conservation may be influenced by small codon usage biases or perhaps other selective effects, which are known to perturb silent site conservation in several other species [24]. In any case, any such selective pressures acting on this presumed neutral sequence will make our functional predictions more conservative.

### Conserved Intergenic Blocks in P. chabaudi, P. berghei, and P. yoelii

To determine intergenic regions likely to be functional, we searched regions upstream and downstream of orthologous mouse malaria genes for aligned blocks with strong conservation. The three mouse malaria species were generally well-aligned in both the 5' and 3' regions. In the 300 bp upstream of gene translation starts, the fraction of sites with the same base in all three species was 0.51, and in the 300 bp downstream of the translation stop the conservation rate was also 0.51. The conservation rate was somewhat lower (0.39) for the full 5' sequences up to 2500 bp. This suggests the prevalence of functional sequence closer to the gene, as has been observed in yeast and mammals [see also Additional file 2].

To detect blocks of unusually conserved sequence among the three mouse malaria species, we identified 40 bp windows with a conservation score above a threshold level (see Methods). The conservation score was calculated as the sum of contributions for each base within the window identical across all three species. For a given window, the score is calculated as a sum over all identical positions in the alignment *i *of the base-dependent score *σ*(*b*_*i*_). Non-identical positions provide no contribution. In other words, the score *S *is defined as

S=∑iσ(bi)δ(i),
 MathType@MTEF@5@5@+=feaafiart1ev1aaatCvAUfKttLearuWrP9MDH5MBPbIqV92AaeXatLxBI9gBaebbnrfifHhDYfgasaacH8akY=wiFfYdH8Gipec8Eeeu0xXdbba9frFj0=OqFfea0dXdd9vqai=hGuQ8kuc9pgc9s8qqaq=dirpe0xb9q8qiLsFr0=vr0=vr0dc8meaabaqaciaacaGaaeqabaqabeGadaaakeaacqWGtbWucqGH9aqpdaaeqbqaaGGaciab=n8aZjabcIcaOiabdkgaInaaBaaaleaacqWGPbqAaeqaaOGaeiykaKcaleaacqWGPbqAaeqaniabggHiLdGccqWF0oazcqGGOaakcqWGPbqAcqGGPaqkcqGGSaalaaa@3E50@

where *δ*(*i*) = 1 if the site is identical across all the species and is 0 otherwise. In practice, because A and T compositions are similar to each other, as are G and C compositions, we use only two values of *σ*, *σ*_AT _and *σ*_GC_. This composition dependence is important in evaluating sequence conservation across malaria genomes, due to their strong AT bias. This scoring approach is quite stringent in its requirement of identity across all the species, which has the advantage of decreasing false positives. While this is a simplification of the information in the phylogeny, alternative methods with a more complex approach to the species' phylogenetic relationships are not noticeably better, and have additional caveats (see Discussion).

We set a threshold score S^min ^such that the multinomial probability of a random sequence having that score or greater was < 10^-6 ^(see Methods), in order to limit the number of false positives. This corresponded to a minimum score S^min ^of 64.0. Since the total amount of alignable *P. yoelii *sequence is 1.5 Mb, we expect ~1.5 false positives.

**Figure 2:**
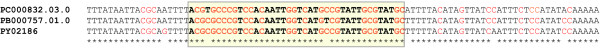
**A 40 bp region upstream of the *P. yoelii *gene PY02186 conserved beyond the neutral background**. The region is shown with corresponding orthologous sequences of *P. chabaudi*, *P. berghei*. The conservation pattern of the highlighted block – in addition to 609 other blocks in the *P. yoelii *genome – has a less than 10^-6 ^probability of occurring by chance, based on silent site substitution patterns. While the unhighlighted sections are mostly conserved as well, the highlighted region stands out for its high density of conserved G's and C's, which are rare (6.3%) in 4-fold degenerate positions.

In total, we observed 610 5' blocks with a score greater than or equal to S^min^. This is 400 times the expected number of false positives, indicating widespread conservation for functional reasons. An example of one strongly aligned region is shown in Figure 2, which shows a region 5' of the gene PY02186, a conserved hypothetical protein. Because the background conservation level is high, the hallmark of this region is the clustering of conserved GC bases, rather than conservation alone.

The average length of the 5' conserved blocks is 61 bp. Their localization is not strong – they are typically located 503 bp from the *P. yoelii *ATG, with a standard deviation of 459 bp. They comprise a total length of 37.2 kb, which corresponds to 2.4% of the length of the alignable *P. yoelii *5' sequence data. In the 3' regions, we find 208 blocks with an average length of 60 bp, comprising 2.4% of the total 3' dataset.

#### Conserved Intergenic Blocks in Alignments including P. falciparum

*P. falciparum *is significantly further diverged from the three mouse malaria species. Only 0.31 of *P. falciparum *bases in the 300 bp upstream of genes are identical across all four species, with the same fraction in 300 bp downstream of genes. Because of this low neutral sequence conservation and the extreme AT content, alignments of the four species are less reliable except in regions with strong conservation. This is an additional reason for focusing the analysis on regions with a stringent conservation p-value (10^-6^).

To detect conserved blocks among the four species, we applied the same method we used for the three species comparisons but with a window size of 15 bp. Setting a p-value of 10^-6 ^corresponds to a cutoff score of 26.5. In the 5' intergenic regions, we find a total of 81 regions exceeding the cutoff. As there are 1.5 Mb of alignable *P. falciparum *sequence, we expect ~1.5 false positives, which is once again much less than the number of observed hits. The conserved blocks cover only 0.1% of the alignable *P. falciparum *sequence. Thus, *P. falciparum *appears to have a substantially lower fraction of shared functional sequence with the mouse species than they share with each other. In the 3' regions, we find 17 conserved blocks. The 5' blocks have an average length of 24 bp, with an average location 451 bp upstream of the *P.f*. start (std. dev. = 425 bp).

These 81 blocks occur upstream of 73 distinct genes and 29 of them have functional annotation. These 29 are shown in Table 1. Several of these genes are involved in ribosomal function, including PF07_0079, PF08_0014, PF11_0313, and PFE0960w. The alignments for these and all other regions are available in the online website.

**Table 1:** List of 29 annotated *P. falciparum *genes. These 29 annotated *P. falciparum *genes have strongly conserved blocks aligned with *P. berghei*, *P. yoelii*, and *P. chabaudi *upstream regions. An additional 44 unannotated gene upstream regions contain strongly conserved blocks as well.

MAL13P1.279	cell division control protein 2 homolog
MAL7P1.26	O-sialoglycoprotein endopeptidase, putative
MAL8P1.140	methionine aminopeptidase, putative
PF07_0079	60S ribosomal protein L11a, putative
PF07_0085	ferrodoxin reductase-like protein
PF07_0091	cell cycle control protein cwf15 homologue
PF08_0014	plastid 50S ribosomal protein, putative
PF08_0129	protein phosphatase, putative
PF10_0149	cysteine – tRNA ligase, putative
PF10_0174	26s proteasome subunit p55, putative
PF10_0271	centrin, putative
PF10_0337	ADP-ribosylation factor-like protein
PF10_0368	dynamin protein, putative
PF11_0313	ribosomal phosphoprotein P0
PF11_0461	rab6
PF13_0034	vacuolar ATP synthase subunit h, putative
PF13_0149	chromatin assembly factor 1 subunit, putative
PF14_0256	exosome complex exonuclease rrp41, putative
PF14_0415	dephospho-CoA kinase, putative
PF14_0437	helicase, truncated, putative
PFA0400c	beta3 proteasome subunit, putative
PFB0550w	peptide chain release factor subunit 1, putative
PFE0175c	unconventional myosin pfm-b
PFE0960w	50S ribosomal subunit protein L14, putative
PFI1665w	uncharacterised trophozoite protein
PFI1670c	vacuolar ATP synthase subunit EC, putative
PFL0255c	uga suppressor tRNA-associated antigenic protein, putative
PFL0385c	blood stage antigen 41-3 precursor
PFL1590c	elongation factor g, putative

**Figure 3:**
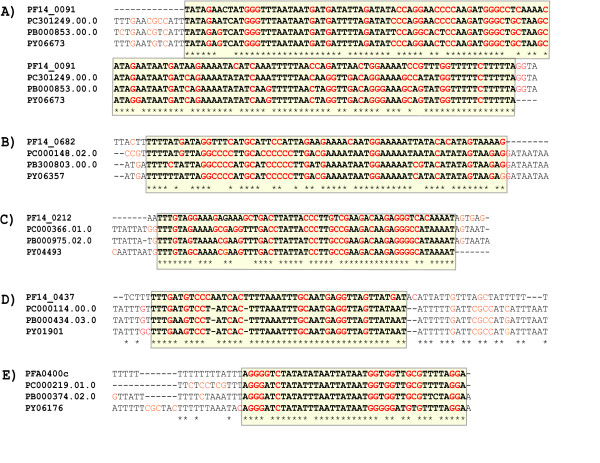
**Upstream blocks conserved beyond neutral expectations among *P. falciparum *and the three mouse malaria species**. These blocks were identified by searching the aligned regions upstream of orthologous genes and are strong candidates for having novel functions. These are (A) at 275 bp upstream of PF14_0091, a hypothetical gene, (B) at 81 bp upstream of PF14_0682, a hypothetical gene, (C) at 185 bp upstream of PF14_0212, a hypothetical gene, (D) at 275 bp upstream of PF14_0437, a helicase truncated putative gene, and (E) at 1 bp upstream of PFA0400c, a beta3 protesome putative gene. In total, we observe 81 *P. falciparum *5' regions with a conservation p-value of 10^-6^or lower.

Five examples of such regions with conservation well beyond the neutral background are shown in Figure 3. These regions are upstream of the unannotated genes PF14_0091, PF14_0682, and PF14_0212, as well as the annotated genes PF14_0437 (a putative truncated helicase) and PFA0400c (a putative beta3 proteasome subunit). As discussed above, the expected fraction of identical sites based on 4-fold positions is ~1/3, and all of these regions are clearly conserved beyond that level. These example blocks are significantly longer than would be expected for a single transcription-factor binding site (approximately 6–12 bp [25,26]), though we also observe some conserved blocks that correspond to the size of a binding site.

We believe that these examples, as well as many others in the set of blocks we have identified, are strong candidates for having novel functional activity. These five blocks each occur within 300 bp of the annotated start of the downstream gene. Typical *P. falciparum *5' UTR lengths are 350 bp (with some examples as long as 990 bp)[27], and some of the conserved blocks are probably in a 5' UTR. Analogous highly conserved sequences have been recently shown to act as enhancers in the vertebrate phylogeny[28], suggesting that these and other strongly conserved regions could be important for *P. falciparum *gene regulation.

**Figure 4:**
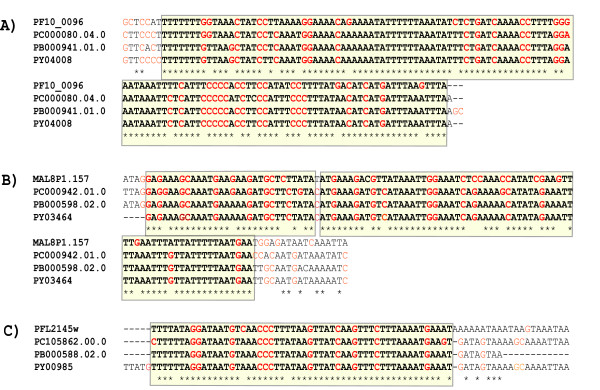
**Downstream blocks conserved beyond neutralexpectations among *P. falciparum *and the three mouse malaria species**. The blocks are located (A) at 172 bps downstream of PF10_0096, a hypothetical gene, (B) at 108 bps downstream of MAL8P1.157, a hypothetical gene, and (C) at 140 bps downstream of PFL2145w, a hypothetical gene. 15 conserved blocks in downstream regions had a p-value of 10^-6 ^or lower.

Three examples of such conserved blocks within 300 bp downstream of a gene are shown in Figure 4. These blocks are adjacent to the hypothetical genes PF10_0096, MAL8P1.157, and PFL2145w. Each of these occurs within the first 200 bp downstream of its respective gene, suggesting the blocks may be in their respective 3' UTRs.

### Most Conserved Blocks Are Not Unknown Coding Sequences or Microsatellites

Although the current database of malaria genes is imperfect, most of the blocks which we have identified do not appear to be previously unknown coding sequences. We BLASTed our conserved blocks against a large dataset of experimentally verified full length cDNA sequences from *P. falciparum *and *P. yoelii *and manually inspected the results. These data sets were downloaded from the Comparasite cDNA database [29]. In the dataset of 610 predicted 5' functional regions across the 3-species, 50 overlap with experimentally validated cDNAs from *P. yoelii*. The number of *P. yoelii *genes which have available cDNA data is 1212 in our dataset, and our complete dataset is made of 3711 orthologs shared across the 3-species. If all the predicted functional regions were in cDNAs we would expect 610 * (1212/3711) = 199 regions to overlap the cDNA data. However, we only observe 1/4 of this number. In our dataset of 81 predicted 5' functional regions across the 4-species, only 2 overlap with experimentally validated cDNAs. There are 2805 4-species gene orthologs in our dataset and 1465 have cDNA data available. So we observe far fewer conserved blocks overlapping cDNAs than would be expected (81 * 1465/2805= 42.3) if all conserved blocks were parts of cDNAs. Note that even if a conserved region overlaps a cDNA it could be in a UTR region rather than an exon, in which case the region's function could still be regulatory.

We have also analyzed the periodicity of the pattern of conservation, which would be expected to have an unusual signal at multiples of 3 nt if the conserved regions were coding sequence. We calculated the autocorrelation of the conservation pattern of the conserved blocks and compared this to the autocorrelation in known exonic regions (see Methods). In the known exonic regions, the autocorrelation value at 3 nt was more than 10% above the value at the neighboring distances (2 nt or 4 nt) in both the 3-species and 4-species data. This was also true at 6 nt, 9 nt, 12 nt, ..., 30 nt. However, this 3 nt periodicity was not observed on average for our predicted functional regions, demonstrating that they are generally not exonic. For the predicted regions, the 3 × nt autocorrelation values were not consistently larger than their neighbors, and were even negative at many of the distances, suggesting that most blocks are not exons [see Additional file 3]. A finer comparison of the autocorrelation pattern of individual 4-species blocks to the idealized exonlike conservation pattern 110110110110... (where 1 and 0 represent a match and mismatch, respectively) did, however, suggest that 11 of the 81 blocks may be exonic [see Additional file 4].

If the predicted conserved regions are important for function, one would expect them to not be made of highly repetitive DNA, which occurs commonly in malaria intergenic regions. To test this, we have run the microsatellite-finding program Tandem Repeats Finder[30] on our conserved 5' regions, using parameters from the previous study of malaria microsatellites by Volkman et al[31]. Microsatellites are predicted in less than 10% of the conserved blocks. In the 610 blocks from the three mouse malariae set, 43 instances of microsatellites are predicted. Furthermore, the predicted microsatellites are different than those that typically occur in malaria intergenic regions. Volkman et al reported that the majority of malaria intergenic microsatellites are repeats of sequences one or two bases long, but the 610 conserved blocks contain only a single instance of 12 consecutive A's and one instance of 12 consecutive T's. Other than that, all the predicted microsatellites occur in at most 4 tandem copies with copy lengths ranging from 4–8 bases. These small numbers are not a side effect of the average conserved block size (61 bp), which is large enough to contain significantly more repeats. For the 4-species set, we observe only 3 predicted instances of microsatellites in 81 total blocks. One of these is an instance of 13 consecutive T's. The other two are repeats that occur 2 and 4.25 times, respectively. These results are consistent with our scoring function having been designed to favor GC-rich blocks, which would exclude many simple repeating units such as microsatellites.

### Conserved Motifs

An alternative approach to identifying conserved blocks is to search for sequence motifs whose instances are conserved unusually often. This approach has been used successfully to identify short functional sites in yeast [11,32] and mammals [12]. Such motif-based methods take advantage of the fact that some types of functional motifs, such as transcription factor binding sites, occur repetitively throughout the genome, and this allows them to be more easily detected. To discover such motifs, we enumerated all N-mers (5 ≤ N ≤ 10) and measured how often they are conserved across the three mouse malaria species.

For each motif of length N, we calculated the fraction of its occurrences in which all three mouse species had the identical motif in the alignment, and we then compared this value to the fraction of all N-nucleotide windows which are identical across the species. For example, the fraction of *P. yoelii *8-bp windows which are identical across all three species is 0.10 (quite close to the expectation from the 4-fold sites of 0.757^8 ^~ 0.11). We used these values and the number of instances of each motif to calculate a conservation z-score z_p_. Gaussian statistics were then used to assign a p-value at given z_p_, and motifs were retained if they satisfied: p-value < 4^-N^. Because of the strong AT content of malaria genomes, we found that some AT-rich motifs occurred in frequencies much higher than would be expected for transcription factor binding sites, and these high frequencies led to excessive values of z_p_. Therefore, we restricted to motifs occurring no more than 1000, but at least 10 times in the genome.

As a second restriction, we calculated another z-score z_n _based on the number of copies of the motif and the expected number given the base composition of the motif and average base frequencies in the *P. yoelii *genome. This additional criterion was applied because we expect that functional motifs should occur more frequently than would a random sequence of comparable base composition. Only motifs with z_n _> 5 (p < 6 × 10^-6^) were retained.

**Figure 5:**
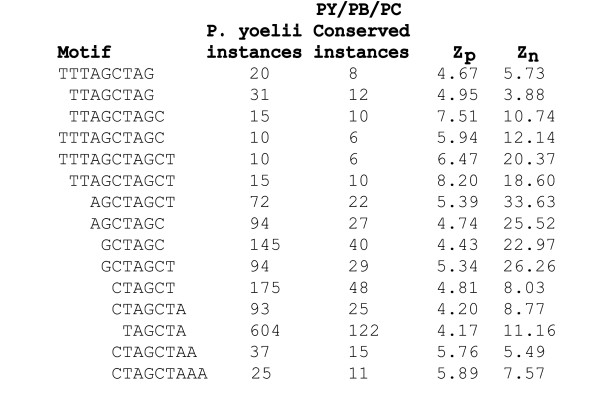
**Variants of the AGCTAGCT motif**. Each of these variants of the AGCTAGCT motif was found to have a statistically significant z-score for conservation across the rodent malaria species (Z_p_) and for overoccurrence in *P. yoelii *(Z_n_). Interestingly, this motif is also its own reverse complement. Of the 33 motifs with significant values of Z_p _and Z_n_, 15 of them cluster by sequence similarity into this family.

In the 5' upstream regions, we found 33 motifs with significant conservation among the three rodent malaria species. These motifs were clustered using MUSCLE [33], resulting in 3 major families, though the motifs in each family were similar enough to be easily clustered by eye as well. The first of these families includes the core motif AGCTAGCT and 14 other similar sequences (Figure 5). The AGCTAGCT motif occurs in 72 instances in the alignable regions and is conserved in 22 of them, whereas one would expect only 7.4 to be conserved by chance (z_p _= 5.4, z_n _= 33.6). It is worth noting that this motif is its own reverse complement, which is often a sign of functional transcription factor binding sites. Other related variants include the motifs TTAGCTAGCT (z_p _= 8.2, z_n _= 18.6) and CTAGCTAA (z_p _= 5.8, z_n _= 5.5). Interestingly, a merging of these sequences suggests an extended motif of TTAGCTAGCTAA, which is also its own reverse complement. Although the AGCTAGCT motif is not conserved in the aligned *P. falciparum *sequence, 4 of the 7 *P. falciparum *genes contain an AGCT motif within 200 bp of the corresponding alignment position, and a fifth gene contains an AGCT 600 bp upstream. These findings suggest that, despite the lack of clear conservation, the corresponding function may have persisted in *P. falciparum*.

There are two additional motif families which make up most of the remaining significant motifs: TGCACAC and TGTGTG. As in the case of AGCTAGCT, each of these motifs has several variants which are also significantly conserved. The TGCACAC motif has 8 additional variants, and the TGTGTG motif has 5 additional variants. These motifs are also nearly reverse complements of one another, suggesting that they correspond to binding sites of the same transcription factor but on opposite DNA strands.

For each of the three motifs AGCTAGCT, TGCACAC, and TGTGTGT, there is a bias to be localized closer to the gene, suggesting the motifs may be involved in gene regulation. This is especially true in the first 400 bp upstream of the translation start [see Additional file 5]. We also analyzed the fraction of these three motifs' instances conserved across the three species, as a function of their distance from the *P. yoelii *translation start (in bins of 100 bp). Although we observed conserved instances of motifs at many different distances, the bin with the highest conservation rate was from 300–400 bp. This was true for each of the three motifs (AGCTAGCT: 61%, TGCACAC: 56%, TGTGTGT: 40%). This is particularly suggestive of gene regulatory function since malaria 5' UTRs have been measured to be on average 346 bp long. [27].

We next performed a motif search in the 300 bp 3' of the genes, as 3' regions have been shown to be binding sites for microRNAs in other species [12]. The evidence for these motifs was not as strong, as there were only 5 distinct motifs. 4 of these appear to be part of the core motif pattern TTTTCGA (z_p _= 4.5, z_n _= 4.9), and the last is the motif TTTGTG (z_p _= 4.5, z_n _= 4.9). Details are given in Additional file 6.

Unfortunately, due to the extreme sequence divergence between *P. falciparum *and the other species, we found no identifiable motifs in a conservation analysis across all four species. Of the motifs meeting the prescribed z-score and copy number cutoffs, none was conserved in more than one instance, making the results unreliable.

Previously, van Noort and Huynen predicted motifs likely to be functional in *P. falciparum *and *P. yoelii *based on a correlation with gene expression and functional classification. They also found a motif similar to our TGTGTG motif (motif 3 of their supplement), and a similar motif (ATGTGTA) was found by applying the FastCompare algorithm [34], which looks for motifs occurring in orthologous promoters but does not require them to be aligned, to *P. falciparum *and *P. yoelii *(M. Llinas, personal communication). Our TGCACAC motif was not observed in the expression analysis, but a variant was found with FastCompare (ATGCACA). Our AGCTAGCT motif was not observed by either of the other methods.

### Conservation of Previously Characterized Functional Sequences

There are several genomic regions with previous experimental evidence for either regulating expression of the downstream gene or for binding to proteins. We surveyed a number of these to determine if they agreed with the conserved regions we identified.

One of the best-characterized functional sequences in malaria intergenic regions is the G-box, which was first observed upstream of the heat shock protein hsp86 (PF07_0029) in *P. falciparum *[3]. In our data, the *P. falciparum *and *P. yoelii *sequences are conserved in the section of the alignment containing two copies of the G-box, which has consensus (A/G)NGGGG(C/A). Unfortunately, there was no sequence for either *P. chabaudi *or *P. berghei *in that region, preventing us from assessing conservation with our 4-species score. However, we calculated an analogous score using just the *P. falciparum *(GCCCCGCGGAAAGGGGC) and *P. yoelii *(GCCCCGTGGTAAGGGGC) sequences. The p-value for their conservation pattern is 10^-29^, indicating extremely strong conservation beyond the background.

The next best example of conservation was for a 120 bp region, in front of the *P. falciparum *dihydrofolate reductase gene, likely to contain sequences controlling gene expression [4]. Although we found no unusually conserved block in the corresponding region using our scoring function, we did observe one 12 bp stretch where all the bases in each species are consistently either purine or pyrimidine (CATTCCATTTA in *P. falciparum*). Crabb and Cowman also experimentally identified a 172 bp region adjacent to the *dhfr *in *P. chabaudi *with regulatory activity [4]. Surprisingly, this region does not contain the same 12 bp purine/pyrimidine pattern and does not align to the functional *P. falciparum *region.

We observed that, in general, most known functional regions do not exhibit conservation between *P. falciparum *and the mouse malariae. We considered several other aligned regions which have been reported to have functional activity in *P. falciparum*, and none exhibited significant sequence conservation. These include the characterized regions of the *DNA Polδ *promoter [7], "Sequence B" in the *PfCDS *promoter [6], and the *PAF-1 *transcription factor binding site in the *pfs25 *promoter [5]. The regulatory region of the *P. falciparum *calmodulin gene [4] was inconclusive, because the previously reported upstream sequence [8] has discrepancies with that currently in PlasmoDB [35]. This overall lack of conservation suggests high turnover in regulatory sites since *P. falciparum *diverged from the other malariae. This is not unexpected given the long divergence times, as evidenced by the saturated K_s _values, between *P. falciparum *and the other species. However, it also indicates that the unusually conserved blocks we have identified among the four species must be under a considerable amount of selective pressure.

## Discussion

We have identified 610 intergenic sequence blocks likely to be conserved for functional reasons in the mouse malaria species, as well as 81 blocks conserved across these species and *P. falciparum*. Our statistical analysis suggests that the majority of these do not encode protein, though a minor fraction may be previously unknown coding sequences. We have used stringent p-value cutoffs to predict these regions (p < 10^-6^), making them strong candidates for being functional. Given the small number of known intergenic functional sites in malaria, this high stringency approach should have the most practical utility. Many of our predicted functional blocks have even higher stringency. For example, at a required p-value of 10^-9^, we still find 20 4-species blocks meeting the cutoff.

In addition we have identified 3 recurrent motifs, AGCTAGCT, TGCACAC, and TGTGTG, with strong evidence for being functional in the mouse malaria species, based on both conservation and overrepresentation. One caveat is that these motifs are only mildly conserved with *P. falciparum*, and it is unclear whether the gene regulation pattern would be the same in all four species. In general, most of the known *P. falciparum *regulatory regions in our dataset are not conserved with the mouse malariae, which is likely due to the large divergence time between *P. falciparum *and the other species. Intriguingly, a recent study has shown that a TGCAC motif is functional in *P. falciparum *[36].

We have estimated that 2–3% of the alignable sequence is functionally conserved among the mouse malariae, and ~0.1% is functionally conserved among the 4 species. Both of these numbers are considerably less than the estimates for the amount of functional intergenic sequence shared among *sensu stricto *yeasts [10] (~30% of the alignable sequence, ~90 bp per promoter) or for mammals [37,38] (2× as much conserved non-coding sequence as coding sequence). This result is consistent with previous suggestions that transcription regulation may be less important in malaria than in other eukaryotes [16]. A mitigating consideration is that *P. falciparum *is at saturated divergence from the other malaria species. This makes it difficult to estimate the total amount of functional intergenic sequence in *P. falciparum*, since some may be functional but under different selective constraint than in the rodent malaria species. However, this mitigating factor should be less relevant when only the rodent malariae are considered, since they are much more closely related. For the aforementioned yeasts, the fraction of identical 4-fold sites is 0.33[10], while for *P. yoelii*-*P. berghei*-*P. chabaudi*, the fraction of identical 4-fold sites is 0.757. So the 3 malaria species are more similar at their 4-fold sites than the yeast species, yet have a lower fraction of constrained intergenic sequence. One other caveat is that the methods used to predict the amount of functional sequence in the yeast and mammalian phylogenies are not identical to ours, though they are similarly based on the concept of calibrating versus neutrally evolving sequence.

The small amount of predicted conserved functional sequence is not an artifact of our stringently set score cutoff. At each possible cutoff score, we measured the percentage of sequence in conserved blocks and subtracted the estimated percentage of false positive sequence (the p-value multiplied by the typical block length at that cutoff score). For the 4-species comparison, this quantity has a maximum value at approximately 1% (cutoff score ~57), which is much less than what is observed for the *sensu stricto *yeasts. A similar calculation for the three mouse malariae yielded a maximum value of ~9%, which is also significantly less than the yeast estimate of 30%.

For the *P. falciparum *analysis, we have restricted our predictions to regions with sequence in all four species, making it a conservative estimate. However, there do not appear to be many additional regions conserved between *P. falciparum *and only one mouse malaria species. We found 81 blocks using all 4 species, but only 15 more conserved blocks in comparisons between *P. falciparum *and *P. yoelii*, which has significantly more sequence data than the two remaining species. Adding the contributions from all of these comparisons would not be sufficient to bring the fraction of unusually conserved sequence up to that of yeast.

Regional inversions and transpositions do not appear to have significantly affected our efficacy in detecting functional sequences. We performed a BLAST local alignment of all *P. yoelii *and *P. falciparum *orthologous promoters. We found only 4 additional conserved blocks from such local alignments (e-value < 10^-6^), which is again only a minor perturbation to the total amount of predicted functional sequence.

One interesting question is how the different minimum length requirements of the 4-species and 3-species methods affect the predictions. Among the 81 blocks conserved across four Plasmodium species, 21 overlap with the 610 conserved in the three species. (Note that even though the 3-species method detects only 1/4 of the 4-species predicted blocks, adjustment by this factor would still yield less functional predictions than the yeasts – which have 10× as much predicted functional sequence.) The partial disjointness of the predictions suggests that the minimum lengths give differences in the abilities to detect different types of functional sequence. Qualitatively, we expect the 15 bp method to be better at detecting short functional sequences (e.g. single transcription factor binding sites), while the 40 bp method will be better at detecting longer sequences with a diffuse conservation signal (e.g. clusters of weak transcription factor binding sites and RNAs). The 40 bp method may also detect some short sequences provided they have a strong enough conservation signal (e.g. a highly conserved GC-rich transcription factor binding site). Conversely the 15 bp method may detect binding site clusters and RNAs provided those regions contain at least one shorter block that is sufficiently conserved.

Empirically, we have observed that the amount of predicted sequence is relatively stable to the block size. For example, in the 5' predictions for the 4-species, we have analyzed block sizes of 15 bp, 20 bp, 40 bp, and 60 bp. For this range of sizes, the number of predicted blocks ranges from 81 to 109. For the 3-species analysis, the number of predicted blocks has a somewhat broader range but of a consistent order of magnitude, from 282 to 781.

There is some evidence for codon usage selection in malaria; however we do not believe that these effects are strong enough to substantially alter the neutral model we have inferred from the 4-fold sites. This is suggested by analogy with yeast. For example, Chin et al [10] decomposed selective and neutral pressures on silent sites in yeast, using a combination of information from substitution rate distributions, functional annotations, and codon usage. We have reanalyzed that dataset, observing that the decomposed neutral substitution rate (0.67) differs by only 3% from the overall 4-fold substitution rate (0.65). These decomposed yeast 4-fold site substitution rates were successfully used as a calibration for detecting known transcription factor binding sites, which supports the use of 4-fold sites as a neutral background in malaria as well. In *P. falciparum*, Peixoto et al showed that GC content at the 3rd codon position correlates positively with gene expression [39]. Nevertheless, AT-ending codons still dominate virtually all genes. There are only 18 *P. falciparum *genes with a 3rd codon position GC content of over 40%, and the intergenic GC content (15%) is comparable to synonymous site GC content (17%) [22]. While codon usage selection is not completely understood in malaria, these facts suggest that silent sites are mostly neutral.

Finally, we would like to comment on our block evaluation method. While several alternative algorithms to identifying conserved regions exist, ours has advantages over each of them for studying malaria. One is to build a predictive log-odds statistic for functional regions by contrasting a training set of functional regions against neutral sequence [40]. Unfortunately, because of the paucity of known functional intergenic sequences, this approach is not possible in malaria. A second approach is a phylogenetic hidden Markov model. We applied one such model, phastCONS [37], to our data. However we observed that phastCONS had difficulty resolving many obvious conserved blocks, such as those in Figure 3, even under a variety of reasonable choices of input parameters (functional block size, fraction of protein coding regions predicted to be functional, etc.). phastCONS makes use of phylogenetic distances, but this feature does not appear to have much benefit in the malaria phylogeny due to the close grouping of the three mouse malariae and their extreme divergence from *P. falciparum*. A third algorithm that has shown promise in vertebrate phylogenies is a composition-independent scoring of conservation patterns with respect to neutral expectations, with p-values assigned by Karlin-Altschul statistics [41]. However, for a strongly compositionally biased genome such as malaria, it is critical to distinguish between AT and GC sites, since they have extremely different conservation statistics. We have implemented a method similar in spirit to this third approach, but making the important correction for compositional effects. Another valuable feature of our method is its calculation of p-values based on multinomial statistics, which are more exact than Karlin-Altschul statistics. Based on these considerations, we believe our method is the most appropriate for analyzing selective conservation in the malaria phylogeny.

## Conclusion

We have identified a large number of strongly conserved intergenic regions in malaria species which are excellent candidates for regulatory function. Given the tiny number of previously studied regulatory sequences, this is a significant improvement on the previous knowledge. We have built a database to provide all sequence alignments and predictions of functional sequence, which will be valuable to researchers with interest in any of the individual regions. From a broader perspective, our results indicate that less malaria intergenic sequence has been conserved by selection than in yeast or vertebrate genomes. This supports the hypothesis that transcriptional regulation is simpler in malaria than other eukaryotic species.

## Methods

Published genomic sequence data for the four species *P. berghei*, *P. chabaudi*, *P. yoelii *and *P. falciparum *were downloaded from PlasmoDB [35]. Gene locations for *P. chabaudi *and *P. berghei *were obtained via the Sanger center, using the annotations reported by Hall et al [14]. We calculated a set of orthologs using a reciprocal best BLASTP criterion for these genes. The results were largely in agreement with the annotations from Hall et al. For consistency, the results in this paper are based on the Hall et al ortholog set. We produced alignments of regions 5' of the annotated translation start and 3' of the annotated translation stop site for each gene. For the 5' sequences, up to 2500 basepairs of sequence were taken, with the sequence truncated at any adjacent gene or contig break. This inclusive length was chosen because some known enhancer elements have been observed as far as 1600 bp upstream of *Plasmodium *genes [6]. For the 3' sequences, up to 300 basepairs of sequence were aligned, which is comparable to sizes analyzed in 3' UTR studies in yeast [42]. Similar 5' and 3' lengths have been used in previous studies of malaria as well [22,43]. Alignments were produced first for the three mouse malaria species *P. yoelii*, *P. berghei*, and *P. chabaudi *using MUSCLE [33], and another set of alignments was produced using these three species together with *P. falciparum*. In total, we analyzed 3711 gene ortholog triplets between *P. chabaudi*, *P. berghei*, and *P. yoelii*, and 2805 quadruplets including *P. falciparum*.

Our sequence data are based on 3× coverage of the *P. berghei *and *P. chabaudi *genomes, 5× coverage of the *P. yoelii *genome, and essentially complete coverage of the *P. falciparum *genome. While contigs for *P. berghei *and *P. chabaudi *are shorter than for the other species, we were still able to extract a considerable amount of intergenic sequence. In a multiple-species sequence alignment, there may be regions where a gap character occurs in at least one species. Since not all of the species have fully sequenced genomes, this gap may occur either because there is an indel, or because one of the species has not yet been sequenced in the region. We considered only portions of the alignment for which each species had at least one sequenced base 5' to the region in question, and at least one sequenced base 3'. Because of this restriction, gaps in the alignment should correspond to insertions or deletions, rather than regions which have not yet been sequenced. Such indels contain information about sequence differences across species, while sequencing gaps do not.

After this restriction, the amount of alignable sequence per promoter was 359 bp (PB), 316 bp (PC), and 474 bp (PY) for the 3 way alignments, considering only those (3248) orthologs with non-zero amounts of sequence. For the 2544 4-way alignments, the numbers were 360 bp (PB), 318 bp (PC), 475 bp (PY), and 605 bp (PF). The amount of alignable sequence in tails was 157 bp (PB), 148 bp (PC), and 159 bp (PY) for the 3280 3 way alignments, and 159 bp (PB), 148 bp (PC), 163 bp (PY), and 166 bp (PF) for the 2580 4 way alignments.

For the alignment of coding sequence, sequences were translated, aligned at the amino acid level, and then back-translated to produce alignments at the DNA level. Silent substitutions were then calculated using 4-fold degenerate codon positions. These provide a homogeneous set of bases under relatively weak selection and which are likely to be aligned correctly. To ensure equivalent sequence context we restricted the 4-fold degenerate positions to those in which the preceding two bases and subsequent base were identical in all the species, and we excluded data from any genes containing fewer than 10 alignable 4-fold sites.

### Detection of Conserved Blocks

In the comparison of the 3 mouse species, we analyzed 40-bp windows and evaluated a score for each window. The score was calculated based on the number of A or T bases identical across all the species and the number of identical G or C bases, with non-identical bases giving no contribution. i.e. the score was set to S = n_AT_σ_AT _+ n_GC_σ_GC_. This separation into G/C or A/T bases is crucial in malaria because of the strong AT bias in the genome. For a given species, the values of σ_AT _and σ_GC _are set equal to -log_10_((f_A _+ f_T_)/2) and -log_10 _((f_C _+ f_G_)/2), respectively. f_A_, f_T_, f_C _and f_G _represent the frequency of A, T, C and G bases in upstream untranslated sequences up to 2500 bp. In this computation, we have used only the upstream sequences and not the downstream untranslated sequences to avoid double counting of bases where they overlap. Since the base compositions of each species are similar but not identical [15], the overall values of σ_AT _and σ_GC _are the sum of their respective values in each species. Thus S is a score function based on the logarithm of the likelihood of observing a particular pattern of conserved sites. The species-specific base compositions are given in Additional file 7.

To evaluate the statistical significance of a given score, we calculated the exact probability of observing a random intergenic block of 40 bp with score greater than the observed value. For random blocks, the probabilities of an identical G/C base, an identical A/T base, and non-identical base were set equal to their respective frequencies at shared 4-fold sites, and each site was assumed to be independent. The p-value was calculated using a multinomial expansion of the quantity (p_GC _+ p_AT _+ q)^40^, where p_GC _is the frequency of identical G or C bases, p_AT _is the frequency of identical A or T bases, and q is the frequency of non-identical bases. A sum of the contributions of terms with score greater than that of the given sequence was calculated using Mathematica.

Windows with p-value < 10^-6 ^were accepted. This p-value was chosen in order to make the expected number of false positives approximately one. Because the aligned sequence data is 1.5 Mb, we expect ~1.5 false positives at this cutoff threshold. This allows one to infer that the predicted regions are unlikely to be false positives. A similar calculation was done for the 4-species comparison, though using 15 bp windows instead of 40. Detected windows were then extended by accepting any adjacent identical bases, and overlapping blocks were merged. These window sizes were chosen to be just larger than the smallest sizes sufficient to detect regions with p-value of 10^-6 ^or better, since longer window length gives greater discriminating power. Note that the lengths differ in the two sets of comparisons because the 4-species phylogeny has greater phylogenetic divergence, which allows equivalent discriminating power at a shorter window size. As a guide to intuition, our 3-species blocks necessitate at minimum 9 conserved GC's in a 40 bp block to give a p-value of 10^-6 ^or better, though the number of conserved AT bases is also important. The 4-species method requires at minimum 2 conserved GC's in a 15 bp block.

### Block Analysis

The autocorrelation analysis was performed using the ACF and Pearson correlation functions in the R statistical computing environment. An aggregate autocorrelation was calculated for all genes by weighting the autocorrelation value of each single gene by its length, then dividing the result by the total length of all genes. An analogous procedure was applied for determining the aggregate autocorrelation in conserved blocks. The microsatellite analysis was performed using the *P. yoelii *sequence for the 3-species analysis and using *P. falciparum *for the 4-species analysis since these two species have most comprehensive genome data in their ortholog groups.

## Authors' contributions

HI performed the computational experiments, analyzed data, and contributed to the writing of the manuscript. JP developed the online database. JC conceived of the study, analyzed data, and contributed to the writing of the manuscript. All authors have approved the final manuscript.

## Supplementary Material

Additional file 1 Comparative genomics of malaria species. The website with viewable sequence alignments, conserved blocks, conserved blocks overlapping a cDNA sequence, and conserved AGCTAGCT motifs is available at Click here for file

Additional file 2 Conservation vs. distance from the ATG. Average conservation score as a function of distance upstream from the gene, for the three mouse malaria species and the three mouse malaria species and *P. falciparum*. Click here for file

Additional file 3 Conserved blocks do not have the 3 nt autocorrelation that is characteristic of coding regions. Autocorrelation vs. distance (in nucleotides) for genes aligned across the 3-species and across the 4-species and the corresponding autocorrelation patterns for conserved blocks. Click here for file

Additional file 4 A few 4-species blocks have exon-like conservation patterns. Quantification of exon-like conservation patterns of highly conserved blocks. Click here for file

Additional file 5 Distribution of motif locations relative to the background. Distribution of each motif shows that the motif is located close to the gene. Click here for file

Additional file 6 Unusually conserved 3' motifs across the three mouse malaria species. Unusually conserved 3' motifs across the three mouse malaria species and their copy number, conserved instances and statistical significance. Click here for file

Additional file 7 The species specific base composition. The species-specific base compositions calculated from all gene 5' regions of size up to 2500 bp in each respective species. Click here for file
