# Supplementary material for: Sequences conserved by selection across mouse and human malaria species
Source: BMC Genomics. 2007 Oct 15;8:372. doi: 10.1186/1471-2164-8-372 (PMC2174483; doi:10.1186/1471-2164-8-372)
Supplement: Additional file 1 — Comparative genomics of malaria species. The website with viewable sequence alignments, conserved blocks, conserved blocks overlapping a cDNA sequence, and conserved AGCTAGCT motifs is available at [file 1471-2164-8-372-S1.doc]

**Additional file 1: Comparative genomics of malaria species**

The website with viewable sequence alignments, conserved blocks, conserved blocks overlapping a cDNA sequence, and conserved AGCTAGCT motifs is available at <http://bioinformatics.bc.edu/chuanglab/malaria/malaria.html>


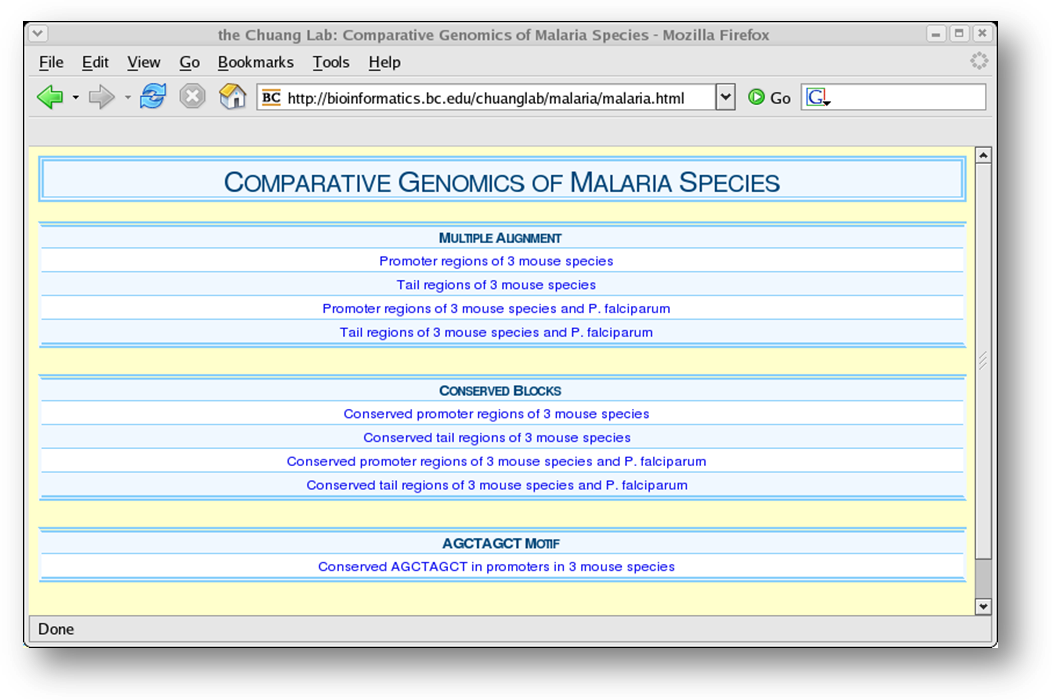


Figure 1. The main web interface of Comparative genomics of malaria species database
