# Supplementary material for: Sequences conserved by selection across mouse and human malaria species
Source: BMC Genomics. 2007 Oct 15;8:372. doi: 10.1186/1471-2164-8-372 (PMC2174483; doi:10.1186/1471-2164-8-372)

**Additional file 2: Conservation vs. distance from the ATG**

Average conservation score (y-axis) as a function of distance upstream from the gene (x-axis), for the three mouse malaria species (A) and the three mouse malaria species and *P. falciparum* (B). The sliding window sizes are 15 and 40, for the former and the latter, respectively. Conservation is strongest close to the gene, but decreases further from the gene. The data have been averaged based on the number of promoters with data at the position. Data was considered to exist at a location if each species had at least one sequenced base 5’ to the region in question, and at least one sequenced base 3’. The large variance past -2500 bp is due to the small number of 5’ regions extending to that length.


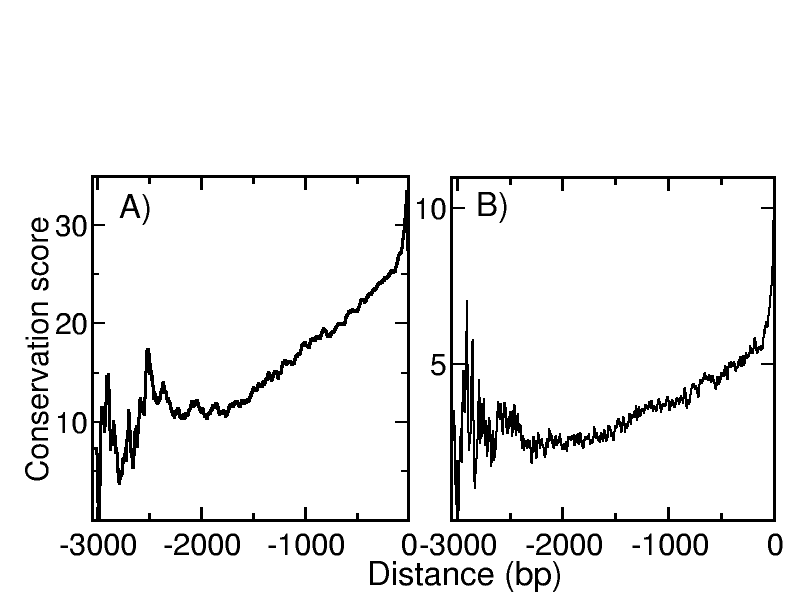

Supplement: Additional file 2 — Conservation vs. distance from the ATG. Average conservation score as a function of distance upstream from the gene, for the three mouse malaria species and the three mouse malaria species and P. falciparum. [file 1471-2164-8-372-S2.doc]
