# Supplementary material for: Sequences conserved by selection across mouse and human malaria species
Source: BMC Genomics. 2007 Oct 15;8:372. doi: 10.1186/1471-2164-8-372 (PMC2174483; doi:10.1186/1471-2164-8-372)
Supplement: Additional file 3 — Conserved blocks do not have the 3 nt autocorrelation that is characteristic of coding regions. Autocorrelation vs. distance (in nucleotides) for genes aligned across the 3-species and across the 4-species and the corresponding autocorrelation patterns for conserved blocks. [file 1471-2164-8-372-S3.doc]

**Additional file 3: Conserved blocks do not have the 3 nt autocorrelation that is characteristic of coding regions**

A) Autocorrelation vs. distance (in nucleotides) for genes aligned across the 3-species. Note the peaks at 3nt, 6nt, 9nt, 12nt, … 30 nt. B) Autocorrelation vs. distance (in nucleotides) for genes aligned across the 4-species. This figure also shows strong peaks at multiples of 3 nt. C) Autocorrelation vs. distance for conserved blocks aligned across the 3-species. There are no peaks at multiples of 3 nt. The strongest peak, which has a considerably smaller magnitude than the peaks in figures A and B, is at 2 nt. D) Autocorrelation vs. distance for conserved blocks aligned across the 4-species. Similarly to figure C, there are not strong peaks at multiples of 3nt, though there is some periodic structure. This is analyzed further in Additional file 4.

**
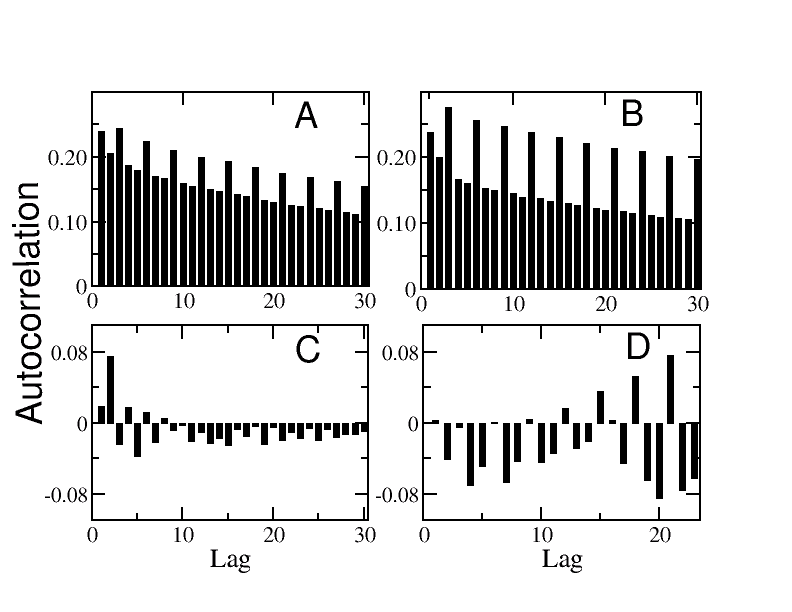
**
