# Supplementary material for: Sequences conserved by selection across mouse and human malaria species
Source: BMC Genomics. 2007 Oct 15;8:372. doi: 10.1186/1471-2164-8-372 (PMC2174483; doi:10.1186/1471-2164-8-372)
Supplement: Additional file 4 — A few 4-species blocks have exon-like conservation patterns. Quantification of exon-like conservation patterns of highly conserved blocks. [file 1471-2164-8-372-S4.doc]

**Additional file 4: A few 4-species blocks have exon-like conservation patterns.**

The autocorrelation of the conserved blocks of 5' regions across 4 species [see Additional file 3 D] shows a weak periodic pattern that is similar to the triplet pattern of coding regions. This appears to be caused by exon-like behavior in a small number of the blocks. To quantify this, we first obtained the autocorrelation of each conserved block. We then compared it to the autocorrelation signal of the ideal triplet pattern 110110110... where 1 and 0 correspond to a matched and mismatched base, respectively. To measure the similarity of the autocorrelation signature of a block and a sequence with an ideal triplet pattern, we calculated the Pearson correlation of these two. These calculations were performed by standard R autocorrelation and correlation functions. The triplet pattern was significant (correlation > 0.2) in 11 conserved blocks while the remaining majority of the conserved blocks did not show a significant triplet pattern.

**
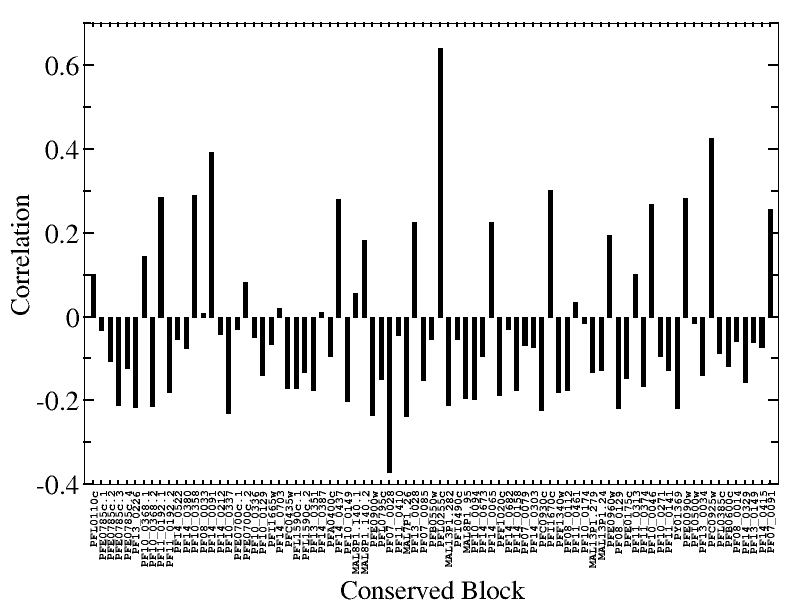
**
