# Supplementary material for: Sequences conserved by selection across mouse and human malaria species
Source: BMC Genomics. 2007 Oct 15;8:372. doi: 10.1186/1471-2164-8-372 (PMC2174483; doi:10.1186/1471-2164-8-372)
Supplement: Additional file 5 — Distribution of motif locations relative to the background. Distribution of each motif shows that the motif is located close to the gene. [file 1471-2164-8-372-S5.doc]

**Additional file 5: Distribution of motif locations relative to the background.**

For each of the three motifs, there is a bias for them to have a localization closer to the gene, consistent with what would be expected if they are functional. For example, in the first 100 bp 5’ to the translation start, all three motifs (AGCTAGCT: 2.6×, TGCACAC: 2×, TGTGTGT: 1.7×) occur considerably more frequently than would be expected by chance, given the lengths of all the *P. yoelii* 5’ sequences in our dataset. Note that the background distribution is not uniform because there is more alignable sequence data available in regions close to the gene.

**
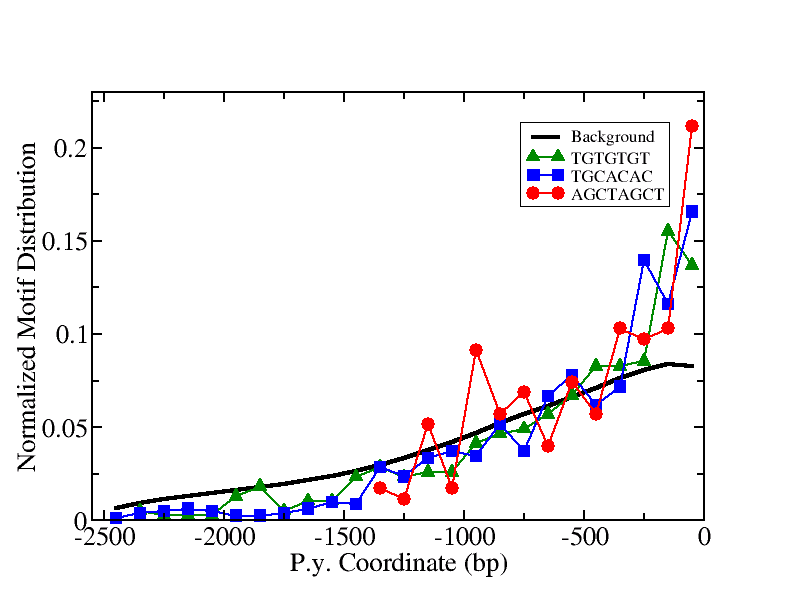
**
