# Supplementary material for: Sequences conserved by selection across mouse and human malaria species
Source: BMC Genomics. 2007 Oct 15;8:372. doi: 10.1186/1471-2164-8-372 (PMC2174483; doi:10.1186/1471-2164-8-372)
Supplement: Additional file 6 — Unusually conserved 3' motifs across the three mouse malaria species. Unusually conserved 3' motifs across the three mouse malaria species and their copy number, conserved instances and statistical significance. [file 1471-2164-8-372-S6.doc]

**Additional file 6: 3’ motifs**

Unusually conserved motifs found in the 300 bp downstream of genes. Conservation is based on the three mouse malaria species, with cutoffs as described in the Methods section.

PY/PB/PC

*P. yoelii* Conserved

Motif Instances Instances z_p_ z_n_

TTTTCG 242 55 3.95167 12.34235

TTTTTCG 146 35 4.47894 15.97393

TTTCGA 157 40 4.12361 3.71219

TTTTCGA 77 22 4.44598 4.93289

TTTGTG 329 69 3.73883 19.38541
