# Supplementary material for: Sequences conserved by selection across mouse and human malaria species
Source: BMC Genomics. 2007 Oct 15;8:372. doi: 10.1186/1471-2164-8-372 (PMC2174483; doi:10.1186/1471-2164-8-372)
Supplement: Additional file 7 — The species specific base composition. The species-specific base compositions calculated from all gene 5' regions of size up to 2500 bp in each respective species. [file 1471-2164-8-372-S7.doc]

**Additional file 7: Base composition**

The species-specific base compositions were calculated from all gene 5’ regions of size up to 2500 bp in each respective species. This included 5’ sequences of genes without orthologs in other species. These composition values were used to calculate the values of σ used in the scoring function, as described in the Methods.

*Plasmodium berghei*: A=0.39125, T =0.38782, C=0.11020, G=0.11074.
*Plasmodium chabaudi*: A=0.39202, T =0.38280, C=0.11169, G=0.11348.
*Plasmodium yoelii*: A=0.40484, T =0.38705, C=0.10106, G=0.10705.
*Plasmodium falciparum*: A=0.41838, T =0.43326, C=0.06931, G=0.07904.
